# Effect of Continuous Renal Replacement Therapy Using oXiris Hemofilter in a Critically Ill Patient With Sepsis and Acute Respiratory Distress Syndrome

**DOI:** 10.7759/cureus.93732

**Published:** 2025-10-02

**Authors:** Osama Sobh, Hussein Soueidan, Afrah Alatifi, Mohamed T Yassin, Marwa M Elmaghrabi

**Affiliations:** 1 Critical Care Medicine, King Saud Hospital, Unaizah, SAU; 2 Research and Development, Med Surg Solutions, Riyadh, SAU; 3 Botany and Microbiology, King Saud University, Riyadh, SAU; 4 Research, Center of Excellence for Research in Regenerative Medicine and Applications, Alexandria University, Alexandria, EGY; 5 Microbiology, King Saud University, Riyadh, SAU

**Keywords:** absorbing filters, acute kidney injury, blood purification, continuous renal replacement therapy (crrt), critical illness, fluid balance, oxiris hemofilter, septic shock

## Abstract

Acute respiratory distress syndrome (ARDS) and sepsis represent severe and often life-threatening conditions, especially in critically ill patients with multiple comorbidities. This case report details the management of a 59-year-old male patient with a complicated medical history, including ischemic heart disease (IHD), hypertension (HTN), diabetes mellitus (DM), and chronic kidney disease (CKD). The patient was admitted to the intensive care unit (ICU) due to acutely decompensated heart failure (HF) and subsequent sepsis, leading to ARDS. His treatment involved initiating continuous renal replacement therapy (CRRT) using the oXiris filter (Baxter International Inc., Deerfield, IL, USA), known for its ability to adsorb cytokines. The oXiris filter was employed as part of a multifaceted approach to manage the patient's acute kidney injury (AKI) in the context of septic shock and a cytokine storm. Remarkably, no adverse effects were noted, and the patient demonstrated clinical improvement. This case supports the feasibility and safety of using CRRT with the oXiris filter in septic patients with AKI and a cytokine storm, providing an effective adjunctive treatment strategy. This technique may offer potential benefits in managing critically ill patients with complex comorbid conditions, highlighting its possible role in improving outcomes for similar clinical scenarios.

## Introduction

Sepsis, the leading cause of death in critically ill patients, is a severe multi-organ syndrome triggered by infection and characterized by high morbidity and mortality. According to Sepsis-3 criteria, it involves life-threatening organ dysfunction due to a dysregulated host response to infection [1]. Pathogen invasion initiates a cascade of pro- and anti-inflammatory responses, leading to systemic immune imbalance, abnormal hemodynamics, metabolic dysfunction, and ultimately multiple organ failure [2].

Septic shock is defined by persistent hypotension requiring vasopressors and elevated serum lactate despite fluid resuscitation. Globally, sepsis affected 677.5 per 100,000 people in 2017, causing around 11 million deaths - nearly 20% of all global mortality [3]. Hospital mortality ranges from 15% to 29%, with one-year mortality reaching 34% [4]. Infections mainly originate from the respiratory and gastrointestinal tracts, commonly involving Gram-positive and Gram-negative bacteria and fungi. Septic shock, affecting 10-20% of ICU sepsis patients, has higher mortality: 36-47% in ICU, 38-56% in hospital, and up to 60% at one year [5].

Acute respiratory distress syndrome (ARDS), a clinical syndrome marked by diffuse alveolar damage and acute respiratory failure, often results from sepsis. A multinational study found that approximately 40% of ARDS cases are under-recognized [6]. ARDS is now viewed as a heterogeneous syndrome with varied clinical and pathological features [7]. As the most common and earliest complication of sepsis, ARDS significantly increases mortality, especially in critically ill patients, with rates between 20% and 50% [5,8]. Sepsis-associated ARDS is linked to greater severity and worse outcomes than non-sepsis ARDS [9]. Early identification of at-risk sepsis patients is therefore crucial [10].

Continuous renal replacement therapy (CRRT), widely used in ARDS and various coronavirus-related pneumonias, includes the next-generation oXiris filter (Baxter International Inc., Deerfield, IL, USA), a high-permeability polyacrylonitrile (AN69)-based extracorporeal blood purification (EBP) membrane, which is capable of removing endotoxins and cytokines [11,12].

## Case presentation

Research originality

During the coronavirus disease-2019 (COVID-19) pandemic, the oXiris filter with CRRT was used in cases of “cytokine storm,” which caused ARDS in COVID-19 patients and was the main reason for admission to the ICU [13]. As the oXiris filter gained popularity recently, it has been investigated in multiple studies and case reports. However, there is ongoing discussion regarding whether it provides benefits in the treatment of patients with septic shock [14]. Hence, Wang and colleagues conducted a comprehensive analysis by combining published and ongoing studies to evaluate the influence of the oXiris filter on clinical outcomes [15]. Briefly, all four randomized controlled trials (RCTs) exhibited an uncertain risk of bias. The certainty of evidence for all outcomes remained low or very low, primarily due to the predominant use of observational studies in the original study design, coupled with unclear risk of bias and limited sample sizes in the included RCTs. This was also confirmed by Sanfilippo and colleagues (2021), who reported low-quality methodologies and clinically based evidence [16].

In addition, while the criteria for defining ARDS have evolved, further evolution is necessary to ensure applicability in low-income settings where resources, such as blood gas analysis, may not be widely available [17].

**Figure 1:**
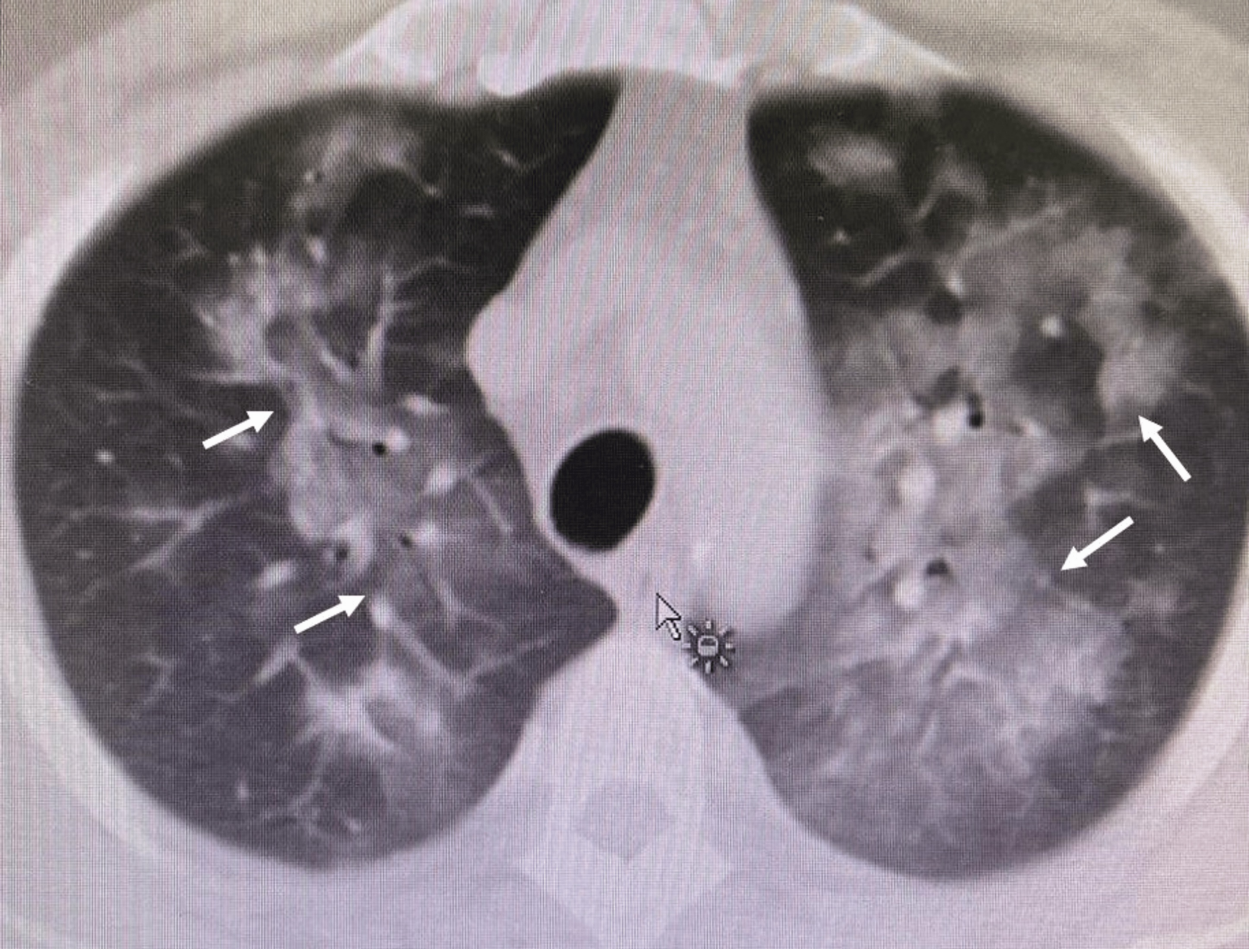
Computed tomography (CT) of the chest showing bilateral lung infection with acute respiratory distress syndrome (ARDS) (arrows).

**Figure 2:**
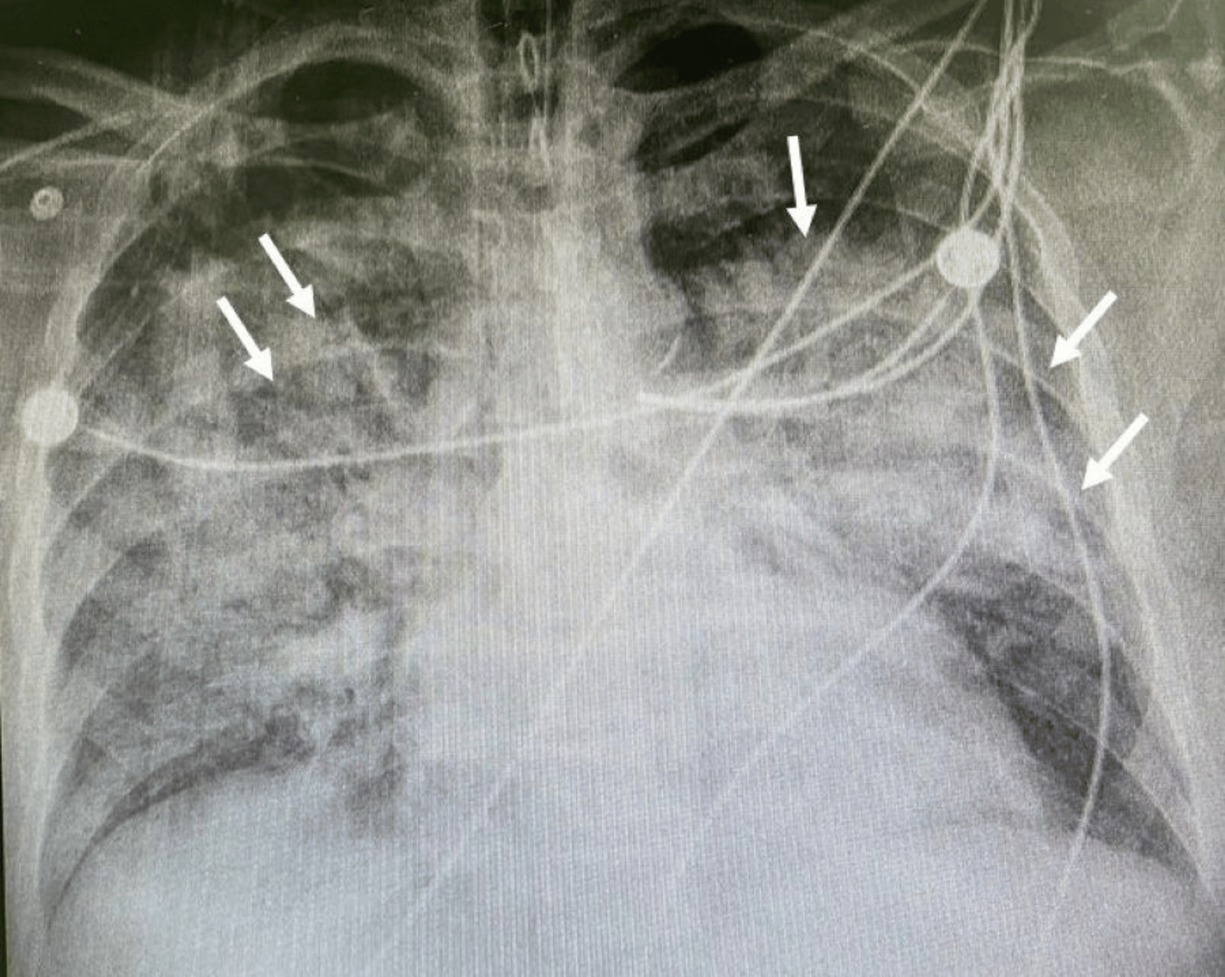
Chest X‑ray showing bilateral lung infiltration with acute respiratory distress syndrome (ARDS) (arrows).

This study outlines a patient case exhibiting complications reminiscent of septic shock associated with COVID-19, where the patient displayed symptoms including ARDS, hypoxemia, and septic shock (Figures 1, 2), suggesting a potential cytokine storm, despite the hospital's inability to directly measure cytokine concentrations, indirect indicators such as ferritin, lactate dehydrogenase (LDH), and C-reactive protein (CRP) markers were utilized. These markers were correlated with the identification of a low-resource setting, as defined. The patient underwent CRRT employing the oXiris filter for 48 hours.

Patient’s history, pathology, and course of treatment

Initial Encounter With the Patient

A 59-year-old male patient presented at the hospital, expressing concern about the gradual onset of shortness of breath (SOB), with no associated chest pain, palpitations, or altered level of consciousness. The patient has a medical history of ischemic heart disease (IHD), hypertension (HTN), diabetes mellitus (DM), and chronic kidney disease (CKD), having undergone percutaneous coronary intervention (PCI) in 2014.

Upon the patient’s admission, blood and septic investigations were performed immediately. As the healthcare team ordered a blood and septic workup, the results were summarized in Table 1.

**Table 1:** Interpretation of the patient’s laboratory results during CRRT with the oXiris filter, showing significant improvement in hemodynamics and sepsis markers. LDH: lactate dehydrogenase (an enzyme marker for tissue damage, e.g., in hemolysis, liver disease, or myocardial infarction); FIO_2_: fraction of inspired oxygen (the concentration of oxygen a patient is breathing, e.g., 0.21 for room air, 1.0 for 100% oxygen); PEEP: positive end-expiratory pressure (a ventilator setting to keep alveoli open at the end of expiration); SpO_2_: peripheral oxygen saturation (oxygen saturation measured by pulse oximetry); GCS score: Glasgow Coma Scale score (assesses level of consciousness (range 3-15)); Hb: hemoglobin; WBCs: white blood cells; HCT: hematocrit; BUN: blood urea nitrogen; ALT: alanine transaminase; AST: aspartate aminotransferase; PTT: partial thromboplastin time; PT: prothrombin time; INR: international normalized ratio; pH: potential hydrogen; PaO_2_: partial pressure of oxygen (oxygen levels in arterial blood, measured via ABG); PaCO_2_: partial pressure of carbon dioxide (CO_2_ levels in arterial blood, indicates ventilation status); HCO_3_^-^: bicarbonate (a key buffer in blood, reflects the metabolic component of acid-base balance); BE: base excess (indicates the amount of acid or base needed to normalize blood PH); Sed and Par: sedated and paralyzed

Variable	Reference Range (Adults)	Day 0	Day 1	Day 2	Day 3	Day 4	Day 5	Day 6	Day 7	Day 8	Day 9	Day 10
Ferritin (ng/mL)	12-300	320	582	-	508	350	309	263	299	355	507	405
LDH (U/L)	140-280	752	634	-	-	471	459	387	518	-	-	439
Temperature (°C)	36.4-37.2	37.2	36.5	36.7	36.7	37.3	36.6	37	37.4	37.2	37	37
Blood pressure (mm Hg)	90/60-120/80	138/56	152/85	151/87	122/74	123/76	139/71	138.62	125/72	128/79	122/82	122/72
Heart rate (beats per minute)	60-100	112	96	79	82	101	103	106	103	102	94	100
Respiratory rate (breaths per minute)	12-16	24	21	18	18	20	20	20	16	18	18	20
Mechanical ventilator	-	Yes	Yes	Yes	Yes	Yes	Yes	Yes	Yes	Yes	Yes	Yes
FiO_2_	21	100	90	80	50	50	40	40	40	40	40	30
PEEP	-	16	13	12	11	8	7	7	6	6	6	6
SpO_2_ (%)	>95	89	91	95	95	97	97	98	98	98	97	98
GCS score	>8	Sed and Par	Sed and Par	Sed and Par	Sed and Par	Sed and Par	Sed and Par	Sed and Par	Sed and Par	Sed and Par	Sed and Par	Sed and Par
24-h urine output (mL)	800-2000	1750	2715	3060	1405	1290	2485	2030	1950	2090	3100	1290
Hb (g/dL)	14-18	8.2	9.4	10.3	9	7.8	7.8	7	7.7	8	8.1	7.9
Platelet count (×10^3^/µL)	135-317	236	231	222	245	188	144	112	77	85	52	69
WBC (×10^3^/µL)	5-10	10.1	14.4	7	15.3	31.7	41.7	28.6	27.7	32.1	26.9	25.7
HCT (%)	40-54	26.7	30.5	32.6	29.3	25.4	25.3	22.5	24.3	25.5	26.4	24.6
Creatinine (mmol/L)	65-119	520	330	203	230	365	250	373	426	424	239	278
BUN (mmol/L)	2.1-8.5	34.8	23.6	19.4	26.6	40	30.4	41	56	39.1	21.5	19
ALT (IU/L)	29-33	231	309	284	236	195	120	77	52	52	43.5	35.1
AST (U/L)	8-33	255	148	89	79	105	45.4	29.1	27.3	33.8	33.6	31.8
Total bilirubin (mmol/L)	1.71-20.5	7.4	8.4	11	14	14	15	15.1	16.1	15.6	18.5	22.8
PTT (seconds)	25-35	36.2	34.3	38.9	38.8	39	43.5	75.5	46.2	63	39.5	32.9
PT (seconds)	11-13.5	15.6	17.7	18.5	17.8	17.8	17.1	18.4	14.9	17.3	11.6	15.7
INR	-	1.2	1.4	1.5	1.45	1.45	1.39	1.37	1.21	1.16	0.98	1.78
Sodium	135-145	145	140	138	139	143	138	138	139	141	142	140
Potassium (mmoI/L)	3.6-5.2	4.1	3.92	2.93	3.73	3.33	3.33	3.61	3.74	3.23	3.02	3.57
Chloride (mmol/L)	96-106	102	99	104	105	102	99	98	98	100	99	100
Phosphorus (mg/dL)	2.8-4.5	2.31	1.75	0.97	1.12	1.82	1.68	2.27	3.02	2.3	1.35	1.38
Albumin (g/dL)	3.4-5.4	25.4	27.8	34	30	30	25.4	23.7	24.8	29.6	30.8	30.4
Lactate (mg/dL)	4.8-25.7	2.2	2.5	2.2	2.3	3.3	2.3	2.3	1.64	1.3	1.8	1.4
pH	7.53-7.45	7.2	7.32	7.41	7.36	7.25	7.35	7.4	7.37	7.32	7.36	7.4
PaO_2_ (mm Hg)	75-100	36	38	22	43.9	44	39	40.3	42	39	39.1	45
PaCO_2_ (mm Hg)	35-45	57	47	31.6	40.7	44	39	40.3	42	39	39.1	45
HCO_3_^-^ (mmol/L)	23-29	19.2	24.2	21.6	22.2	19.7	23.2	20.6	20	20.1	21.5	19.8
BE	-	-3.7	-2	-3	-1.9	-7.1	-2.3	-4.2	-5.9	-6	-6	-5.2
Noradrenaline (mcg/kg/min)	0	20	15	10	5	2	-	-	-	-	-	-
Dopamine (mcg/kg/min)	0	20	15	10	5	2	2	-	-	-	-	-
Modified SOFA	<3	13	12	11	10	11	8	8	8	7	6	6

The clinical examination revealed an oriented and conscious individual experiencing respiratory distress. The echocardiogram indicated impaired left ventricle (LV) systolic function, with a left ventricular ejection fraction (LVEF) of 34% and anterolateral hypokinesia. The mitral valve displayed normal morphology with mild mitral valve regurgitation (MR), while the aortic valve (AV) exhibited normal trileaflet function. The tricuspid valve functioned normally but with moderate tricuspid regurgitation (TR) and a pulmonary arterial systolic pressure (PASP) of 60 mmHg. Additionally, no intracardiac masses or pericardial effusion were observed.

Patient’s Transfer to the Cardiac Care Unit (CCU)

Due to acutely decompensated heart failure (HF), the patient was admitted to the CCU, where he still showed SOB and developed three fever spikes that reached 38^°^C. The chest X-ray imaging revealed patchy infiltration, leading to empiric antibiotic treatment with a Tazocin injection of 4.5 grams every eight hours, a linezolid injection of 600 mg every 12 hours, and a tigecycline injection of 100 mg as a loading dose, followed by 50 mg every 12 hours. The team started vasopressor support and respiratory assistance through non-invasive ventilation (NIV). Despite interventions, the patient continued to exhibit low urine output (UOP).

The patient continues to exhibit unstable hemodynamics, with oxygen saturation (SpO_2_) ranging from 88% to 92%, and elevated white blood cell (WBC) and lactate levels. By the third day in the CCU, the patient's condition worsened, prompting the activation of a rapid response team. As the patient’s SpO_2_ declined, he entered a shock state, and the response team opted for elective intubation and admission to the intensive care unit (ICU).

Patient’s Transfer to the ICU

Upon ICU arrival, CRRT with the oXiris filter was initiated. The patient was put on a mechanical ventilator with high settings (fraction of inspired oxygen (FiO_2_) of 100%, positive end-expiratory pressure (PEEP) of 1 cmH_2_O, and SpO_2_ of 87-89%). He received a noradrenaline (NE) infusion (50 mcg/min) and a dopamine infusion (10 mcg/kg/min), while his initial blood gas analysis indicated metabolic acidosis with a UOP of 10-20 mL/hr.

Results

**Figure 3:**
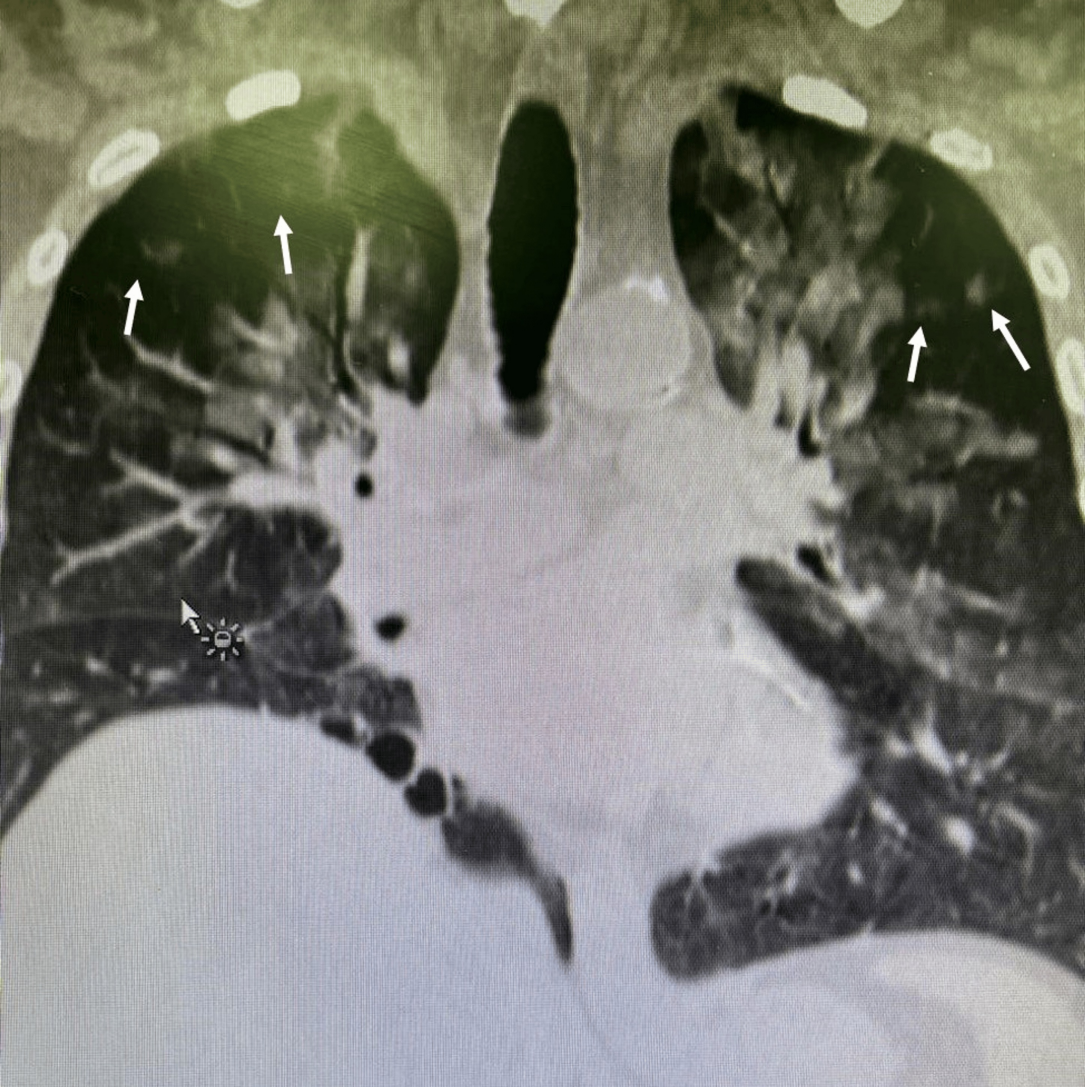
Chest X-ray showing resolution of both lungs after implementation of the treatment protocol (arrows).

During the first 72 hours, the patient's condition improved significantly (Table 1 from D3), showing better hemodynamics and requiring less functional support. SpO_2_ rose to 98%. The demand for support notably decreased due to observed improvements in clinical and hemodynamic status (Figure 3), leading to the decision to initiate weaning and proceed with extubation.

## Discussion

The principal outcome of this case series study suggests that CRRT employing the adsorbing membrane oXiris appears to be clinically viable, devoid of adverse effects, and potentially beneficial for septic patients experiencing acute kidney injury (AKI) alongside a cytokine storm. These findings corroborate certain experimental investigations wherein the oXiris filter demonstrated improvements in renal function and mitigated the inflammatory response in cases of sepsis and AKI [3], as we elaborate here. The swift decline in severe patient conditions is closely linked to cytokine storm-induced sepsis or septic shock, which are primary contributors to mortality among critical patients [18]. In recent years, continuous blood purification therapy has emerged as a therapeutic modality for sepsis.

Multiple studies have explored the effects of the oXiris filter in CRRT. For example, Zhang and colleagues utilized this filter in treating COVID-19 patients. Their findings indicated that continuous blood purification therapy demonstrated potential in reducing cytokine overexpression, stabilizing hemodynamic status, and facilitating gradual improvements in organ function. They concluded that the use of the oXiris filter led to a decrease in endotoxin levels, whereas they remained stable when employing the standard hemofilter [19]. Furthermore, levels of tumour necrosis factor-alpha (TNF-α), interleukins (IL)-6 and IL-8, and interferon γ decreased significantly with the oXiris filter. On another front, Shum and colleagues investigated the progression of Sequential Organ Failure Assessment (SOFA) scores during continuous venovenous hemodiafiltration (CVVHF) with the oXiris filter, comparing it to a matched historical cohort treated with standard CVVHF [20]. They noted a significant decrease of 36% in SOFA scores 48 hours post-initiation of oXiris-CVVHF. In contrast, the historical controls showed a slight increment of 3%, although no disparities in mortality were observed between the two cohorts [21].

In a retrospective analysis involving 60 patients with sepsis or septic shock undergoing continuous venovenous hemodialysis (CVVHD) with the oXiris membrane, improvements in hemodynamics, reduced vasoactive requirements, and decreased SOFA scores within the initial 48 hours of CVVHD were documented. Concurrently, reductions were noted in the levels of IL-6, IL-10, procalcitonin, and endotoxin activity. However, mortality data for the cohort were not provided, and it remains uncertain whether some patients had succumbed before the completion of the 48 hours of CRRT [17].

In a separate retrospective cohort study encompassing 30 patients with septic shock and AKI who received CRRT with the oXiris membrane between 2014 and 2019 across two French centers, researchers observed a hospital mortality rate lower than anticipated for patients with the highest severity of illness according to Simplified Acute Physiology Scores (SAPS). However, no reduction in SOFA scores during the initial 48 hours of CRRT with oXiris was detected. The oXiris hemofilter, specifically engineered for endotoxin adsorption, introduces an innovative approach by leveraging the inherent hydrogel structure of the AN69 membrane. Furthermore, the surface of the oXiris filter is augmented with coatings of polyethyleneimine (PEI) and heparin [17]. PEI, characterized by a multilayer linear structure and cationic properties, demonstrates the capability to adsorb endotoxins bearing negative charges on its surface. Simultaneously, the negatively charged moieties within the bottom layer can sequester inflammatory cytokines [21]. Previous studies have indicated that the application of PEI on surfaces can enhance the endotoxin adsorption and cytokine removal efficacy of the AN69 membrane. Notably, in comparison to alternative hemofilters, the oXiris hemofilter exhibits notable efficacy in the elimination of inflammatory mediators and adsorption of endotoxins.

Although some studies did not find oXiris beneficial in their context, like in the case of Kang and colleagues [21], our study confirms the other end of the spectrum. To solve this argument, we resorted to systematic reviews about oXiris using the PubMed database, resulting in two papers [15], although neither addresses our specific condition, using the filter in ARDS patients. However, it is insightful to mention that Sanfilippo and colleagues identified very low levels of evidence that were not sufficient to reach a solid conclusion [16]. Iba and Fowler (2017) concluded that administration of the oXiris filter during CRRT in septic patients could potentially correlate with reduced 28-day, 7-day, and 14-day mortalities, as well as lower lactate levels, SOFA scores, NE dosage, and shorter durations of ICU stays [13]. Nonetheless, owing to the limited or very limited quality of evidence, the efficacy of oXiris filters remained uncertain. Furthermore, no significant disparities were noted concerning 90-day mortality, ICU and hospital mortalities, or the length of hospital stay. But at the same time, it is important to mention that the study acknowledged the limitations of such conclusions due to access to data, small sample size, and single-center design of each. Therefore, the authors recognized the need for larger RCTs to confirm the conclusions against the efficacy of the oXiris filter [15].

Beyond our case, it is important to mention that in case the patient's case continues to deteriorate, the physician can reassess the management protocol, including the membrane choice, epinephrine dose, supportive therapy, and multiple organ therapy.

Limitations and recommendations

This report has several shortcomings that are worth mentioning. First, we included only one patient, making the results specific to a person, although they concur with other studies' findings. Second, we have no control group, making it impossible to draw confident conclusions regarding the filter's efficacy in this case. Third, our limited resources at the hospital prevented the measurement of cytokines and mediators, so we resorted to the measurement of other biomarkers such as ferritin, LDH, and CRP. Finally, the literature reports that the filter might also capture antibiotics in addition to cytokines, which may have also slowed down his recovery.

It is recommended to reinvestigate the efficacy of the oXiris filter in a wide sample of patients who suffer from septic shock with a variety of comorbidities across numerous large-scale hospitals through RCTs.

## Conclusions

Hemoperfusion with the oXiris membrane effectively reduced vasopressor support, inflammatory markers, and endotoxin concentrations in patients with refractory septic shock. This approach may offer a novel strategy for early immune modulation in sepsis before renal dysfunction occurs. Further studies with larger cohorts are required to validate these findings and determine optimal treatment protocols.

## References

[1] Singer M, Deutschman CS, Seymour CW (2016). The Third International Consensus definitions for sepsis and septic shock (Sepsis-3). JAMA.

[2] Shankar-Hari M, Harrison DA, Rubenfeld GD, Rowan K (2017). Epidemiology of sepsis and septic shock in critical care units: comparison between sepsis-2 and sepsis-3 populations using a national critical care database. Br J Anaesth.

[3] Rudd KE, Johnson SC, Agesa KM (2020). Global, regional, and national sepsis incidence and mortality, 1990-2017: analysis for the Global Burden of Disease Study. Lancet.

[4] Laupland KB, Zygun DA, Doig CJ, Bagshaw SM, Svenson LW, Fick GH (2005). One-year mortality of bloodstream infection-associated sepsis and septic shock among patients presenting to a regional critical care system. Intensive Care Med.

[5] Schwindenhammer V, Girardot T, Chaulier K (2019). oXiris® use in septic shock: experience of two French centres. Blood Purif.

[6] Bellani G, Laffey JG, Pham T (2016). Epidemiology, patterns of care, and mortality for patients with acute respiratory distress syndrome in intensive care units in 50 countries. JAMA.

[7] Thille AW, Peñuelas O, Lorente JA (2017). Predictors of diffuse alveolar damage in patients with acute respiratory distress syndrome: a retrospective analysis of clinical autopsies. Crit Care.

[8] Wang H, Huang J, Liao W (2021). Prognostic value of the red cell distribution width in patients with sepsis-induced acute respiratory distress syndrome: a retrospective cohort study. Dis Markers.

[9] Zhao J, Tan Y, Wang L, Shi Y (2020). Discriminatory ability and prognostic evaluation of presepsin for sepsis-related acute respiratory distress syndrome. Sci Rep.

[10] Battaglini D, Fazzini B, Silva PL (2023). Challenges in ARDS definition, management, and identification of effective personalized therapies. J Clin Med.

[11] Al-Dorzi HM, Aldawood AS, Khan R (2016). The critical care response to a hospital outbreak of Middle East respiratory syndrome coronavirus (MERS-CoV) infection: an observational study. Ann Intensive Care.

[12] Case J, Khan S, Khalid R, Khan A (2013). Epidemiology of acute kidney injury in the intensive care unit. Crit Care Res Pract.

[13] Iba T, Fowler L (2017). Is polymyxin B-immobilized fiber column ineffective for septic shock? A discussion on the press release for EUPHRATES trial. J Intensive Care.

[14] Zhang L, Cove M, Nguyen BG (2021). Adsorptive hemofiltration for sepsis management: expert recommendations based on the Asia Pacific experience. Chin Med J (Engl).

[15] Wang G, He Y, Guo Q (2023). Continuous renal replacement therapy with the adsorptive oXiris filter may be associated with the lower 28-day mortality in sepsis: a systematic review and meta-analysis. Crit Care.

[16] Sanfilippo F, Martucci G, La Via L (2021). Hemoperfusion and blood purification strategies in patients with COVID-19: a systematic review. Artif Organs.

[17] Turani F, Barchetta R, Falco M, Busatti S, Weltert L (2019). Continuous renal replacement therapy with the adsorbing filter oXiris in septic patients: a case series. Blood Purif.

[18] Fleischmann C, Scherag A, Adhikari NK (2016). Assessment of global incidence and mortality of hospital-treated sepsis. Current estimates and limitations. Am J Respir Crit Care Med.

[19] Zhang H, Zhu G, Yan L, Lu Y, Fang Q, Shao F (2020). The absorbing filter Oxiris in severe coronavirus disease 2019 patients: a case series. Artif Organs.

[20] Shum HP, Yan WW, Chan TM (2016). Extracorporeal blood purification for sepsis. Hong Kong Med J.

[21] Kang K, Luo Y, Gao Y (2022). Continuous renal replacement therapy with oXiris filter may not be an effective resolution to alleviate cytokine release syndrome in non-AKI patients with severe and critical COVID-19. Front Pharmacol.

